# Integrative metabolomic and transcriptomic analyses reveal the mechanisms of Tibetan hulless barley grain coloration

**DOI:** 10.3389/fpls.2022.1038625

**Published:** 2022-10-25

**Authors:** Congping Xu, Hafiz Muhammad Khalid Abbas, Chuansong Zhan, Yuxiao Huang, Sishu Huang, Haizhen Yang, Yulin Wang, Hongjun Yuan, Jie Luo, Xingquan Zeng

**Affiliations:** ^1^ State Key Laboratory of Hulless Barley and Yak Germplasm Resources and Genetic Improvement, Lhasa, China; ^2^ Tibet Academy of Agricultural and Animal Husbandry Sciences, Lhasa, China; ^3^ School of Life Science and Technology, Wuhan Polytechnic University, Wuhan, China; ^4^ Sanya Nanfan Research Institute of Hainan university, Hainan Yazhou Bay Seed Laboratory, Sanya, China

**Keywords:** qingke, flavonoid, anthocyanin, gene network, transcription factor

## Abstract

Cereal grains accumulate anthocyanin during developmental process. The anthocyanin content increases at grain filling stages to develop grain coloration in cereals. However, anthocyanin biosynthesis responsible for grain coloring and its regulatory mechanisms controlled by structural and functional genes remain unclear. Therefore, this study aimed to explore the global map of metabolic changes linked to grain coloration of Tibetan hulless barley (qingke) using an integrative metabolome and transcriptome approach. Grains from three colored qingke cultivars at different developmental stages were considered for molecular and metabolic investigations. A total of 120 differentially accumulated metabolites (DAMs) and 8,327 differentially expressed genes (DEGs) were filtered. DEGs were mainly enriched in the phenylpropanoid and flavonoid pathways. The transcript levels of anthocyanin biosynthesis genes (*PAL*, *C4H*, *4CL*, *CHS*, *FLS*, *F3H*, *F3’H*, *DFR*, *ANS*, *GT*, *OMT*, and *MAT*) significantly upregulate in colored qingke compared to the non-colored variety. During grain development and maturation, the strong correlation of *HvMYC2* expression with anthocyanin contents and anthocyanin biosynthesis genes suggested it as a critical gene in anthocyanin accumulation. Further results confirmed that *HvMYC2* could be activated by *HvMYB* and be a positive regulator of UV-B and cold tolerance in qingke. In addition, verification based on enzymatic assays indicated that six key modifier enzymes could catalyze glycosylation, malonylation, and methylation of anthocyanins, thereby dissecting the major anthocyanin modification pathway in colored qingke. Overall, our study provides global insight into anthocyanin accumulation and the mechanism underlying grain coloration in qingke.

## Introduction

Tibetan hulless barley (*Hordeum vulgare* L. var. *nudum*), known as “qingke” in Chinese, is a staple calorie source for the human population in the Tibetan area. It has attracted increasing research attention owing to its unique nutritional value (Guo et al., 2020). Cultivated qingke shows different grain coat colors, such as white, blue, and purple. Recently, the demand for colored qingke grain as a functional food has increased because of its higher levels of bioactive compounds, such as anthocyanins, compared to white qingke (Bellido and Beta, 2009; Yao et al., 2022). Anthocyanins have essential health properties that reduce the incidence of cancer, cardiovascular disease, and other chronic diseases (Hatier et al., 2008). In addition, anthocyanins can protect qingke against UV light and drought and defend it from invasion by pathogens (Zeng et al., 2020; Xu et al., 2021; Xu et al., 2022).

Anthocyanin biosynthesis shares a common upstream pathway with the flavonoid pathway. These upstream genes include phenylalanine ammonia-lyase (*PAL*), flavanone 3-hydroxylase (*F3H*), 4-coumarate: CoA ligase (*4CL*), cinnamic acid 4-hydroxylase (*C4H*), chalcone synthase (*CHS*), and chalcone isomerase (*CHI*). Dihydroflavonol-4-reductase (*DFR*), leucoanthocyanidin dioxygenase (*LDOX*), and UDP-glucose flavonoid 3-O-glucosyltransferase (*UFGT*) are specific to the anthocyanin pathway. Various transcription factors, including *MYB*, *MYC*, basic-helix-loop-helix (*bHLH*), and *WD40* classes, were also involved in controlling anthocyanin biosynthesis and accumulation and have already been studied (Ramsay and Glover, 2005; Li et al., 2018). Moreover, anthocyanins are modified by various catalyzing enzymes, such as glycosyltransferase, acyltransferase, and methyltransferase, which confer their stability, solubility, and coloration properties (Wang et al., 2019). Increasing evidences have shown that the accumulation of different decorations of anthocyanins offers plants with high tolerance against abiotic and biotic stress (Wang et al., 2019; Xu et al., 2021). To date, the core network of anthocyanin biosynthesis is conserved over higher plants and has been well characterized (Perez de Souza et al., 2020). However, the enzymes responsible for different anthocyanin modifications still need to be investigated in crops such as qingke.

In recent years, technical advancements in transcriptome and metabolome analyses have provided highly effective ways to identify new genes and metabolites and understand the underlying molecular mechanism of plant organ coloration (Wang et al., 2020; Yao et al., 2022). Integrating transcriptomic and metabolomic analyses in wheat has revealed that variations in anthocyanin content are highly linked to the differential expression of key structural genes, such as *TaCHS* and *TaANS*, in the anthocyanin biosynthesis pathways (Wang et al., 2021). Yao et al. (2022) also identified a series of structural genes and transcription factors in anthocyanin pathways showing the differential expression between purple and white qingke seeds.

To investigate the mechanisms of coloration formation during the grain development process, we established the Grain Metabolic Regulation Network (GMRN), a multitemporal distributed metabolome and transcriptome dataset from three colored grains of qingke varieties at three stages. Using GMRN, we elucidated the anthocyanin biosynthesis pathway and identified the new transcription factors (TFs) controlling the flavonoid biosynthesis. The data from this study improves our understanding of qingke grain color formation and provides valuable information for the genetic improvement of qingke.

## Materials and methods

### Plant material and sample preparation

**Figure 1:**
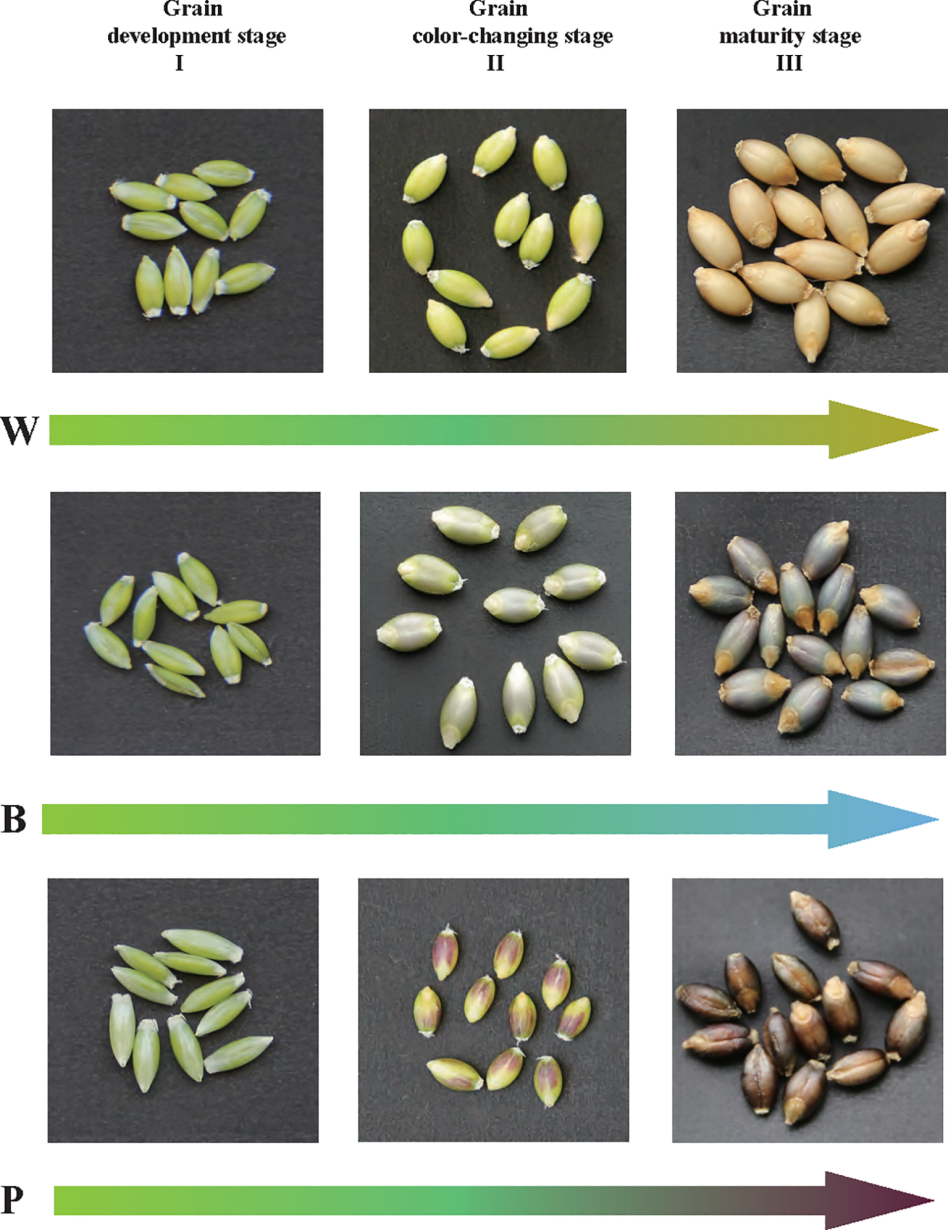
Grains of white (W), blue (B), and purple (P) qingke were analyzed at the grain developmental stage (I), grain color changing stage (II), and grain maturation stage (III).

Three qingke varieties with different seed colors, including purple qingke ‘Zang 0123’ (marked as P), blue qingke ‘Zang 0119’ (marked as B), and white qingke ‘Zang 0131’ (marked as W), were used in this study. Seeds were grown at the experimental station of Tibet Academy of Agriculture and Animal Husbandry Sciences, Lhasa, China. Seeds of the three varieties were collected at different developmental stages of 80, 100, and 120 days after sowing (DAS). These three stages represent the key developmental phases of qingke seeds, including the grain developmental stage (I), grain color-changing stage (II), and grain maturity stage (III) (**Figure 1**). Collected seeds were frozen immediately in liquid nitrogen and stored at -80°C until further experimentation. Three independent biological replicates were used for metabolic and transcriptomic analyses for each sample (at least 30 seeds of each replication).

### UV-B exposure and cold stress

To provide insights into the metabolic responses of two colored qingke varieties (purple qingke ‘Zang 0688’ marked as ‘P1’ and white qingke ‘Zang 0244’ marked as ‘W1’) under UV-B exposure and cold stress (4°C). 15-day-old seedlings were treated with UV-B radiation and exposed to 4°C for 0, 1, and 24 h. Then, the leaf samples from treated and control plants were collected, rapidly frozen in liquid nitrogen, and stored at -80°C for further analyses. Each experiment was repeated thrice.

### Metabolome analyses

Extraction and analyses of flavonoids were conducted as previously described (Shi et al., 2020). Briefly, the freeze-dried seeds were ground into fine powder at 40 Hz for 1.5 mins with a mixer mill (MM400, Retsch, Germany). One hundred milligram of powder was extracted overnight with 1.0 mL of 70% aqueous methanol solution at 4°C, and then centrifuged for 10 min at 10,000×g. The supernatant was aspirated and filtered using a 0.22 µm microporous membrane (SCAA-104, ANPEL, Shanghai, China) and subsequently stored in a vial for LC−MS analysis. Five quality control (QC) samples were prepared by blending all samples equally. Quantification of compounds was performed by scheduled multiple reaction monitoring (MRM) method according to the previous method (Chen et al., 2014). We screened differentially accumulated metabolites (DAMs) with a threshold (|log2fold change (FC)| > 1 and variable importance in projection (VIP) ≥ 1). Principal component analysis (PCA) was performed using SIMCA software 14.1 with default settings.

### RNA extraction and RNA-seq analysis

RNA-seq analysis was conducted as previously (Xu et al., 2021). In brief, clean reads were obtained and aligned to the improved qingke reference genome using HISAT2 software with default parameters (Kim et al., 2015; Zeng et al., 2020). Fragments per kilobase of transcript per million mapped reads (FPKM) were used to calculate the gene expression levels. Differential expressed genes (DEGs) were identified based on the threshold |log2 (FC)| > 1 and *p* < 0.01. We used the pheatmap package of R software to construct hierarchical clustering and heatmaps of DEGs. DEGs were mapped to the KEGG Orthology database and used in the enrichment pathway analysis (Kanehisa et al., 2007). *P* < 0.05 as a threshold for significantly enriched KEGG pathways.

### *K-*means cluster analysis

*K*-means clustering was used to visualize gene expression and metabolite accumulation patterns using MeV (version 4.9.0) as described previously (Eisen, 2002). Normalized expression values of genes and metabolites were calculated using *Z* scores based on input FPKM values of all samples.

### WGCNA and gene network construction

Data were filtered to remove genes that had low expressed in all samples (FPKM <6). A total of 16,759 genes were used to construct co-expression modules using the weighted gene co-expression network analysis (WGCNA) package in R software. Then, four differentially accumulated anthocyanins were imported into the WGCNA package to calculate the correlation between metabolites and gene modules. The correlation coefficient (*r*) range was categorized into 5 correlation classes which included, very weak (*r* 0.0-0.2), weak (*r* 0.2-0.4), moderate (*r* 0.4-0.6), strong (*r* 0.6-0.8) and very strong (*r* 0.8-1.0). The network was visualized using Cytoscape software v3.6.1.

### Quantitative real-time polymerase chain reaction analysis

RNA isolation and cDNA generation were carried out as previously described (Xu et al., 2022). qRT–PCR was performed using the SYBR Green system (TaKaRa, Dalian, China) under the following program: 95°C for 5 min followed by 35 cycles of 94°C for 30 s, 56°C for 30 s, and 72°C for 90 s. A housekeeping gene, *HvUBQ1*, was used as an internal standard for calculating relative gene expression. Quantification was performed using the 2^−ΔΔCT^ method (Livak and Schmittgen, 2001). Specific primers were designed using Beacon Designer™ 7.9 software (Premier Biosoft) (**Supplementary Table 1**).

### Protein expression analysis and enzyme assay

The full-length cDNAs of *HvMAT* and *HvOMT* were cloned into the entry vector pDONR207 (Invitrogen) and then into the expression vector pDEST17 (Invitrogen) by LR recombination. The full-length open reading frames (ORFs) of HvGT1-HvGT4 from P qingke were cloned into the pET30a expression vector with a His-tag. Plasmid extraction was performed with a plasmid extraction kit (Tiangen, Beijing, China). The recombinant plasmid was transformed into *E. coli* BL21 (PG-KJE8) cells. The positive colonies were inoculated into 1 mL of LB medium supplemented with 50 µg/mL ampicillin and 30 µg/mL kanamycin and shaken at 220 rpm for 8 h at 37°C. Five hundred microliters of this inoculum was further grown in 150 mL of LB supplemented with 50 µg/mL ampicillin and 30 µg/mL kanamycin at 37°C to attain an OD_600_ = 0.6, and then 0.4 mM isopropyl-β-D-thiogalactoside (IPTG), and then 25 µg/mL L-arabinose, and 5ng/ml tetracycline were added in for induction at 15°C for 16 h. *E. coli* BL21 (PG-KJE8) cells harboring the empty pET30a (+) vector were used as negative control. Further, cells were collected by centrifugation at 5,000 rpm for 10 mins at 4°C, and the pellets were washed with 20 ml of lysis buffer [NTA: 20 mM Tris, 0.5 M NaCl, (pH 7.9)] and again centrifuged at 5,000 rpm for 10 mins at 4°C. Then, the pellet was resuspended in 2 ml lysis buffer and disrupted by sonication on ice. The resulting homogenate was centrifuged at 12,000 rpm for 50 min at 4°C. The supernatant containing the recombinant His-tagged GT was purified by a HisPur™ Ni-NTA Purification Kit (Thermo Scientific). The imidazole used to elute protein from the Ni-resin was removed by combining fractions containing GT. SDS/PAGE was used to perform protein measurements with BSA as a quantification standard.

*In vitro* enzyme assays were performed using a total volume of 100 µL containing 200 µM substrates, 1.0 mM donors, 100 mM Tris-HCl (pH 7.5), 2 mM dithiothreitol, 10 mM MgCl_2_ and 0.3 µg of the purified proteins. After incubating at 30°C for 20 min, the reaction was stopped by adding 200 µL of ice-cold methanol. The reaction mixture was filtered through 0.2 µm filter (Millipore) before being used for LC−MS analysis.

### Transient expression analysis

For transient expression, the full-length cDNA of *HvMYB2* was first inserted into the entry vector pDONR207 and then into the destination vector pEAQ-HT-DEST2 (GQ497236.1) using the Gateway recombination reaction (Invitrogen). pEAQ-HT-DEST2-*HvMYB2* was transformed into *Agrobacterium tumefaciens* (EHA105). The infiltration mix was injected into the leaves of *N. benthamiana* with a 1 ml syringe without needle. The glyceraldehyde 3-phosphate dehydrogenase (GAPDH) gene of *N. benthamiana* was used as an internal control for comparison.

### Transient dual-luciferase reporter assay

*HvMYC1* and *HvMYC2* were cloned into the pEAQ-HT-DEST vector for the effector construct. The reporter constructs comprised the promoter linked to the reporter gene luciferase. 1000 bp upstream of *HvF3H* was cloned into the pH2GW7.0-Ren-Luc vector *via* a ClonExpress^®^Ultra One Step Cloning Kit (Vazyme, Nanjing). The plasmids were transformed into the *A. tumefaciens* EHA105 strain. After transformation, strains were grown at 28°C for 24 h in LB medium supplemented with 50 mg/L kanamycin and 50 mg/L rifampicillin. Cells were harvested by centrifugation and resuspended in 10 mM MES buffer containing 10 mM MgCl_2_ and 200 μM acetosyringone (Sigma) to a final OD_600_ of 1.2. For coinfiltration, equal volumes of the strains were mixed and infiltrated into *N. benthamiana* leaves. Luciferase activity was determined using the Dual-Luciferase Reporter Assay System (Promega) on a Molecular Devices SpectraMax i3X fluorescence plate reader. All experiments were repeated five times independently.

### Yeast one-hybrid assay

The yeast one-hybrid (Y1H) assay was performed following a previously described method (Zeng et al., 2020). Yeast cells were co-transformed with the pHis2 bait vector harboring the target gene promoter and the pGADT7 prey vector harboring the CDS of *HvMYC2*. As a negative control, yeast cells were transformed with the empty pGADT7 vector and pHIS2 harboring the corresponding promoter. Transformed yeast cells were grown in SD-Leu-Trp medium and SD-Leu-Trp-His medium plates supplemented with 3-AT (Sigma). The plates were incubated for three days at 30°C.

## Results

To investigate and characterize the global changes in metabolic regulatory networks during different grain developmental stages, we created a GMRN dataset from three qingke varieties with different grain color. This dataset contains parallel metabolic profiling and a gene expression matrix for samples (**Figure 1**).

### DAMs among the three colored qingke varieties at three developmental stages

**Figure 2:**
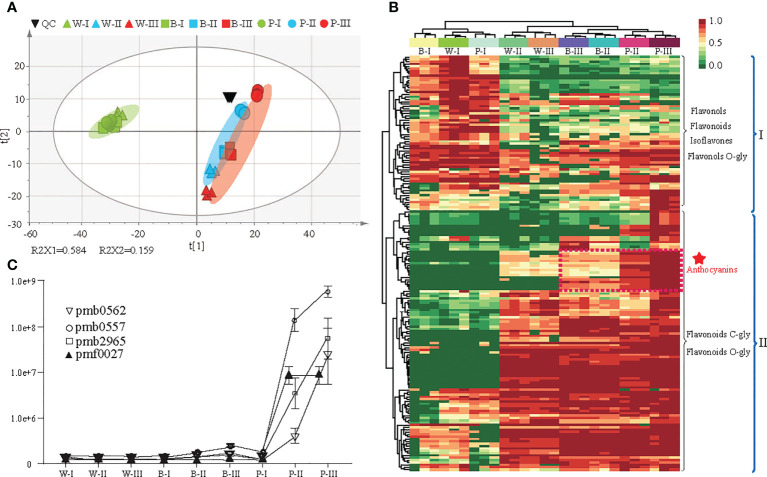
Differential metabolite analysis in three colored qingke varieties at three stages. **(A)** Principal component analysis (PCA) of metabolome data from nine samples. **(B)** Hierarchical clustering heatmap of significant differential metabolites in the flavonoid pathway among the three-color varieties. Metabolites in Clade I accumulate during the grain developmental stage; metabolites in Clade II accumulate during the grain maturity and color-changing stages. The content of each metabolite content was normalized using hierarchical clustering. Red indicates relatively high abundance, and green indicates low abundance. **(C)** Line chart showing the contents of four anthocyanins. Pmb0562 (Pelargonidin 3-(3*”*,6*”*-dimalonyl)glucoside), pmb0557 (Cyanidin 3-(3*”*,6*”*-dimalonyl)glucoside), pmb2965 (Cyanidin 3-(3*”*,6*”*- diacetyl-hexoside)-*O*-glyceric acid), pmf0027 (Cyanidin 3-*O*-galactoside). White (W), blue **(B)**, and purple (P) qingke grains at the grain developmental stage (I), grain color-changing stage (II), and grain maturation stage (III), respectively.

Here, 177 flavonoid metabolites were detected, including 65 flavonoids, 62 flavonols, 25 flavonoid carbonosides, 9 anthocyanins, 9 dihydroflavonols, 4 isoflavones and 3 chalcones (**Supplementary Table 2**). A total of 120 DAMs were identified among the three colored qingke varieties at three developmental stages (**Supplementary Table 3**). Based on all metabolites, principal component analysis (PCA) showed that the quality control (QC) metabolic accumulation patterns were similar, indicating the high quality of our metabolic profiling. Component 1 (R2X1) explained 58.4% of the variability and represented the difference between white- and nonwhite-colored (blue- and purple-colored) samples. Further observations identified that the metabolites in three qingke varieties had similar accumulation patterns at the first developmental stage. However, the accumulation of metabolites had changed significantly at the grain color-changing and maturation stages (**Figure 2A**). In addition, hierarchical clustering analysis (HCA), based on the levels of metabolites, divided these metabolites into two major groups. Fifty-four metabolites in clade I, including flavonols, flavonoids, flavonols O-glycosides, and isoflavones, were enriched during the first stage of grain development. Metabolites in clade II, such as flavonoid C/O-glycosides and anthocyanins, displayed significant overaccumulation during the grain color-changing and maturation stages (**Figure 2B**). Notably, an enormous accumulation of anthocyanins was observed in P variety during the grain color-changing and maturation stages. Four anthocyanins, including pelargonidin 3-(3*’’*,6*’’*-dimalonyl)glucoside (pmb0562), cyanidin 3-(3*’’*,6*’’*-dimalonyl)glucoside (pmb0557), cyanidin 3-(3*’’*,6*’’*-diacetyl-hexoside)-*O*-glyceric acid (pmb2965) and cyanidin 3-*O*-galactoside (pmf0027), accumulated significantly in the purple qingke grains during the grain color-changing and maturation stages (**Figure 2C**).

We divided all 177 flavonoids into ten clusters based on their accumulation patterns using the *k*-means clustering algorithm to gain insight into the metabolic changes in the qingke grains development process (**Supplementary Table 4**). Then, we analyzed the ten clusters and identified metabolites enriched in specific varieties, such as P (Clusters I and III) and B (Cluster VI). We also found metabolites whose contents decreased at the grain developmental stage (Cluster VII) and metabolites whose levels increased (Cluster VIII) at the grain maturation stage. In Cluster V, the contents of several metabolites during the whole grain development process were initially increased and then decreased. Furthermore, several specific compounds (Cluster II) were enriched in colored grains (B and P) during the grain color-changing and maturation stages (**Supplementary Figure 1**).

### DEGs among the three colored qingke varieties during three developmental stages

We conducted transcriptome analysis further to explore the underlying regulation mechanism in color formation. After filtering, the clean reads were mapped to the qingke reference genome. The Q30 percentage (0.02% error rate) was over 90%, and the average GC content across all libraries was 53.7% (**Supplementary Table 5**). Overall, the reads were of high quality and could be used for further analysis. A total of 8,327 differentially expressed genes (DEGs) were filtered among the three colored qingke varieties at the three developmental stages. Specifically, 4,220, 4,123, and 4,104 DEGs were identified at three-grain developmental stages, respectively (**Supplementary Table 6**). KEGG analysis of DEGs revealed that their molecular functions were primarily enriched in phenylpropanoid and flavonoid pathways (**Supplementary Figure 2**).

Principal component analysis (PCA), based on the expression levels of all genes, showed significant and clear clustering for the samples during grain color-changing and maturation stages (**Supplementary Figure 3A**). These genes were also divided into two clusters based on the global expression patterns during the three grain developmental stages. A total of 4,016 genes (22.4%, 4,016 out of 17,908) in Cluster I were highly expressed during the first stage of grain development, whereas 13,891 genes (77.6%, 13,891 out of 17,908) in Cluster II were mainly expressed during the grain color-changing and maturation stages (**Supplementary Figure 3B**).

To further explore the correlations of metabolite accumulation with gene expression patterns, we filtered the genes significantly correlated with at least one metabolite based on a rigorous multiple test correction (*r* > 0.6). Next, 12,306 genes were classified into ten coexpression clusters based on Pearson correlation coefficients, and consistent change in patterns with metabolites were observed. In summary, our results suggested that the patterns of genes were largely in accordance with the dynamics of flavonoid pathway during grain development and maturation stages (**Supplementary Table 7**; **Supplementary Figure 1**).

### Enhancement of flavonoid metabolic flux in blue/purple-colored grains during grain developmental stages

**Figure 3:**
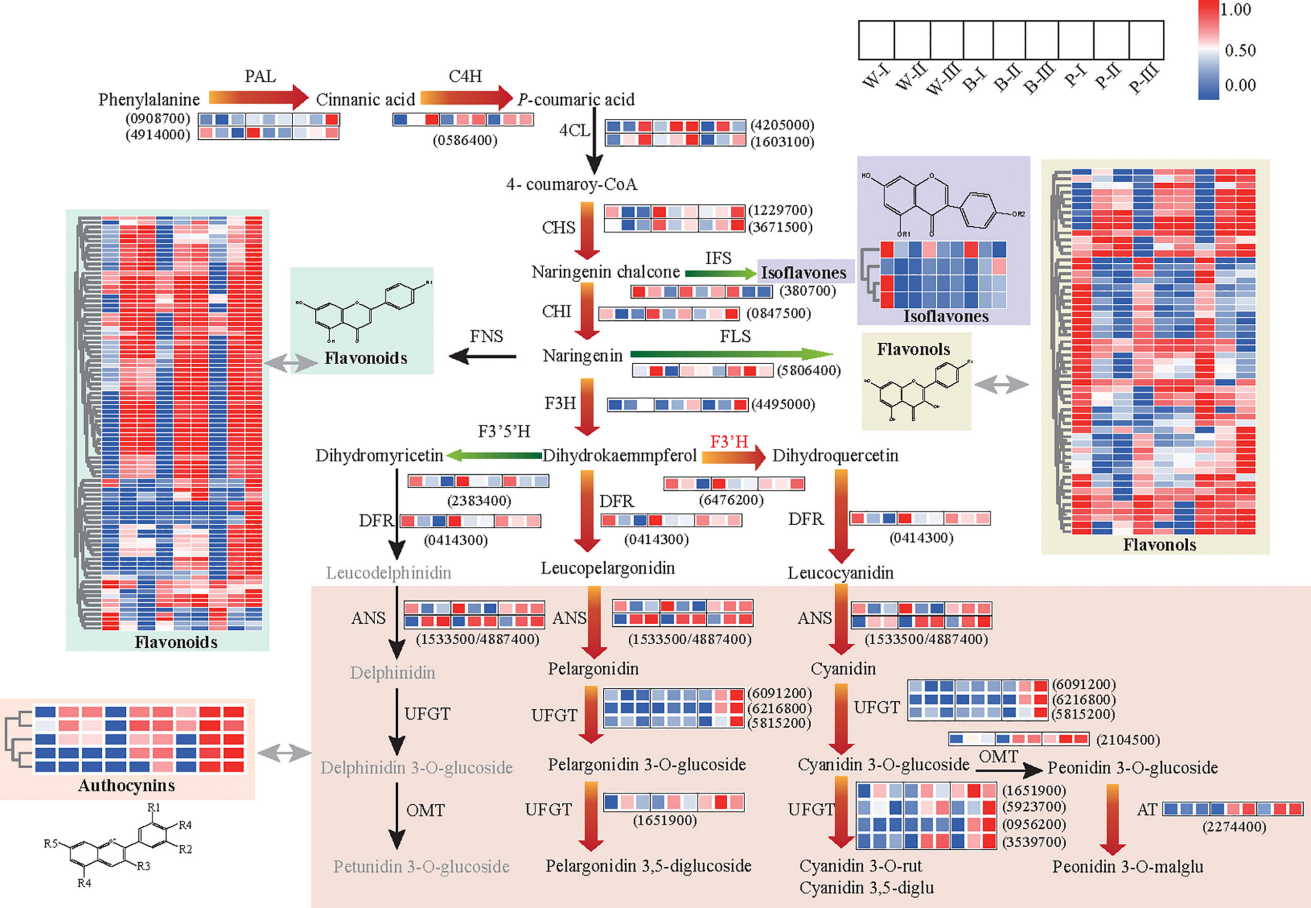
Metabolic pathway for flavonoid biosynthesis and putative transcriptional regulatory network. The heatmap presents the expression of corresponding genes (or the relative contents of metabolites) in the three colored qingke varieties. The color scale from blue to red in the heatmap indicates the FPKM of genes (or the relative contents of metabolites) ranging from low to high. Red arrows indicate the main metabolic flux of colored grains during developmental color-changing and maturation stages. Green arrows represent the direction of the diminished metabolic flux for all varieties during developmental color-changing and maturation stages. The full gene/enzyme names and expression level comparisons are shown in **Supplementary Table 8**. White (W), blue (B), and purple (P) qingke grains in the grain developmental stage (I), grain color-changing stage (II), and grain maturation stage (III), respectively.

To examine whether the GMRN could provide an insight into the dynamic changes in metabolic pathways during grain developmental processes, we first analyzed the differential regulation of metabolites and genes related to flavonoid pathways among three color qingke varieties. At the transcript level, we observed distinct upregulation of central genes (*PAL*, phenylalanine ammonia-lyase; and *C4H*, cinnamate 4-hydroxylase) of the phenylpropanoid pathway, the flavonoid biosynthesis upstream genes (*CHS*, chalcone synthase; *CHI*, chalcone isomerase; and *F3H*, flavanone 3-hydroxylase), switching gene (*F3’H*, flavonoid 3’-hydroxylase) for cyanin biosynthesis and anthocyanin-specific genes (*DRF*, dihydroflavonol reductase; *ANS*, anthocyanidin synthase; *UFGT*, UDP-flavonoid glucosyltransferase; and *AT*, anthocyanin acyltransferase) in colored grains at grain color-changing and maturation stages. In contrast, certain genes, especially those related to the flavonoid and anthocyanin biosynthesis, were downregulated in white color variety at grain maturation stage. In addition, the expression levels of genes related to flavonol branches (*FLS*, flavonol synthase), isoflavone branch (*IFS*, isoflavone synthase), and switching gene (*F3’5’H*, flavonoid 3’5’-hydroxylase) for delphinidin derivatives were significantly downregulated in colored varieties at the grain color-changing and maturation stages (**Figure 3**; **Supplementary Table 8**). The results of quantitative real-time PCR (qRT–PCR) were consistent with the transcriptional data (**Supplementary Figure 4**).

Metabolomics data showed the significant accumulation of phenylpropanoid pathway precursor (naringenin chalcone) and all anthocyanins and their derivatives in colored varieties, especially in purple accessions during the grain color-changing and maturation stages. In addition, we observed a high enrichment of precursors and backbones of flavonoids (naringenin chalcone, butin, catechin, diosmetin, hyperin, isoorientin, jaceosidin) in the first stage of grain development, but various flavonoid glycosides were accumulated constantly, while the contents of the isoflavones and flavonols were significantly decreased during grain development and the maturity stage, regardless of the variety (**Figure 3**). Overall, the above results suggested that the enhancement in flavonoid metabolic flux to increase the accumulation of flavonoids and anthocyanin is highly associated with grain color development in qingke. Our data also revealed a reprogramming of the flavonoid pathway from the isoflavone and flavonol branch toward the downstream branch of flavonoids for all varieties during grain development process.

### Genetic basis for the dynamic changes in anthocyanins during grain development

To gain insight into the genetic basis of color-related metabolites throughout grain development and maturation, WGCNA was conducted to explore the coexpression gene modules associated with anthocyanins. The 7751 genes were divided into 13 gene modules according to their consistent expression trends (**Supplementary Figure 5A**; **Supplementary Table 9**). Next, the contents of four anthocyanins were used as phenotypic data for module-trait correlation. The results showed that the accumulation of transcripts in the salmon module, containing 241 genes, was significantly correlated with four anthocyanins (*r* > 0.6) in three grain developmental stages (**Supplementary Figure 5B**).

**Figure 4:**
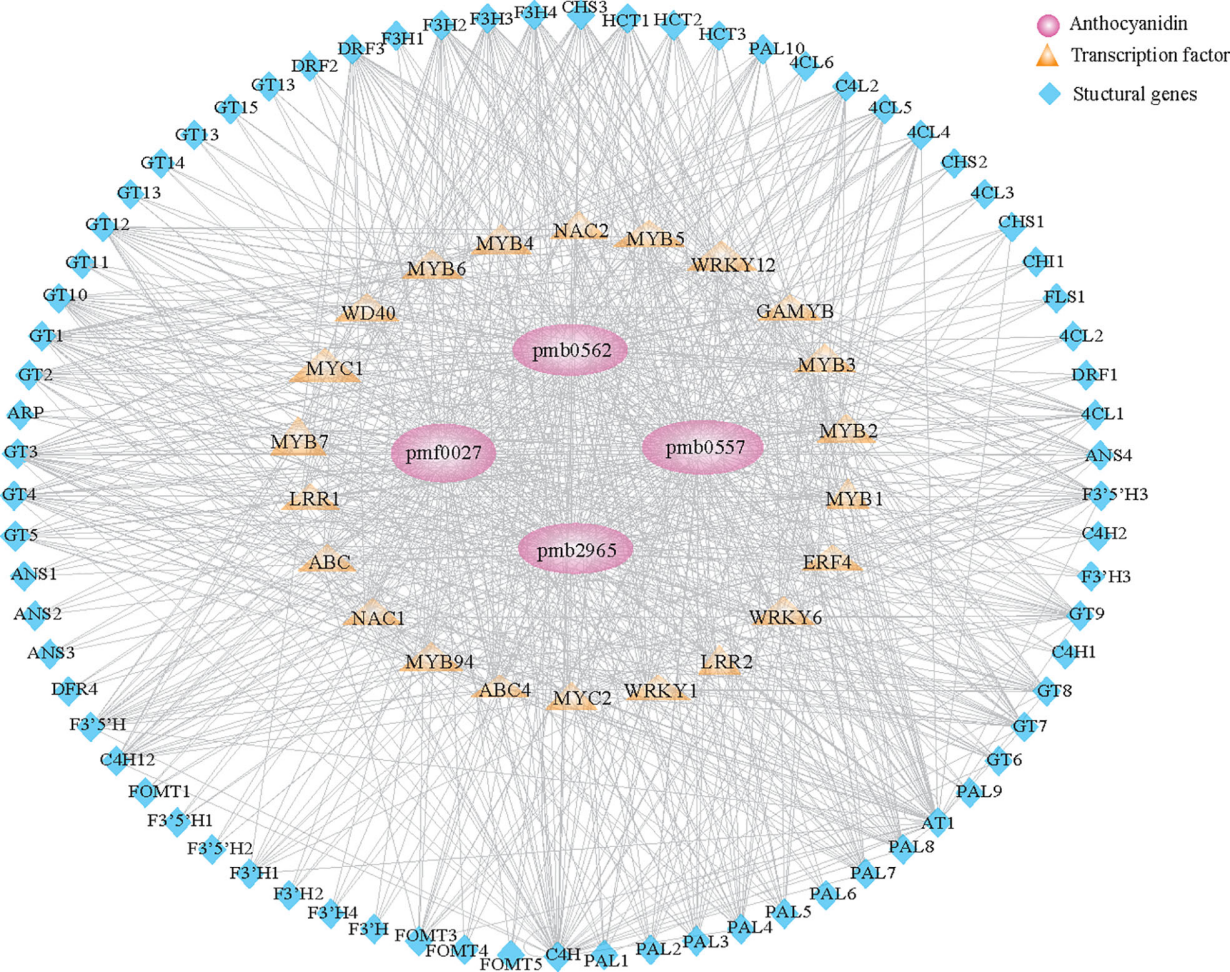
Regulatory network of anthocyanin-related compounds in qingke. Structural genes and transcription factors identified in the salmon module (**Supplementary Figure 5B**) involved in anthocyanin metabolism during the grain developmental stage. Pink circles represent anthocyanins. Orange diamonds represent different families of transcription factors. Blue triangles represent structural genes involved in anthocyanin metabolism.

To construct the regulatory network correlations with anthocyanin metabolism, we screened structural genes involved in the flavonoid pathway in salmon module. Results identified 74 structural genes as suitable candidates, including *PAL*, *C4H*, *4CL*, *HCT*, *CHS*, *CHI*, *F3H*, *DFR*, *ANS*, and *UFGT*, which encode key enzymes in the biosynthesis of anthocyanin. The expression of those identified genes was highly associated (r > 0.6) with the content of anthocyanins. We further constructed the coexpression network based on the identification of 24 transcription factors, including *MYB*, *MYC*, *WD40*, and *ERF*, whose expression was highly associated (r > 0.6) with 74 structural genes (**Figure 4**; **Supplementary Table 10**). Our results suggested that these regulatory genes control anthocyanin biosynthesis in colored varieties throughout grain development.

### Anthocyanin modification pathway

**Figure 5:**
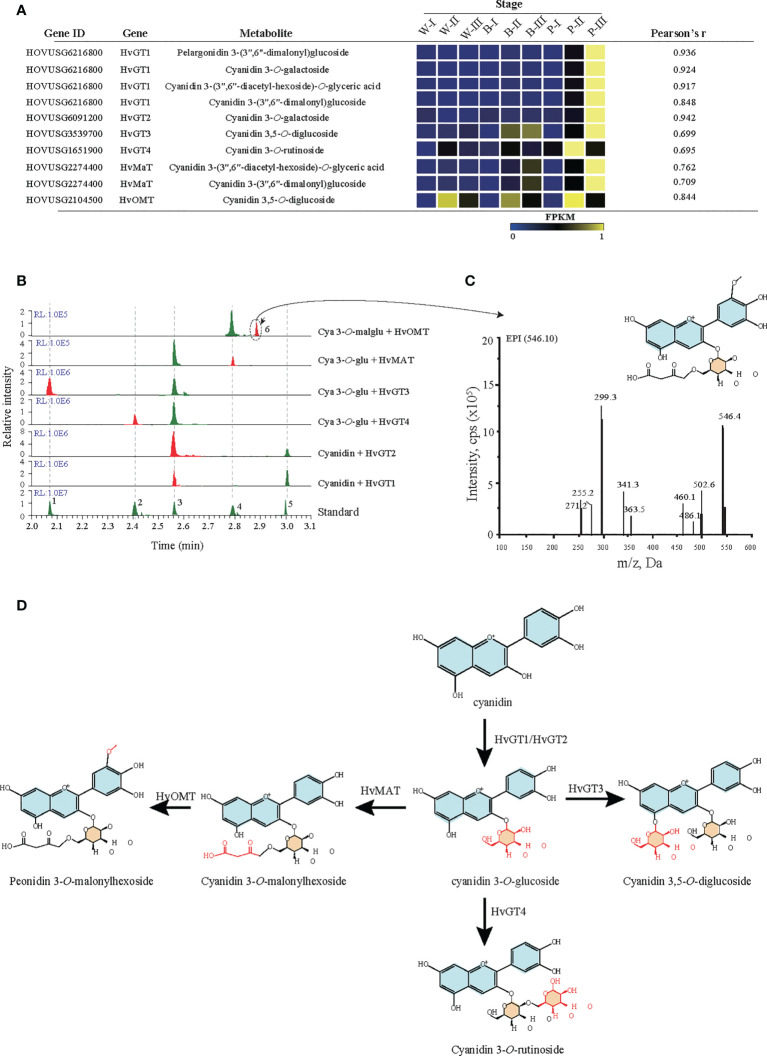
Functional verification of candidate glycosyltransferase, malonyltransferase, and methyltransferase. **(A)** Candidate glycosyltransferase, malonyltransferase, and methyltransferase were identified by Pearson correlation analysis of metabolites and genes. Each candidate gene’s expression represents the fragments per kilobase of transcript per million mapped reads (FPKM). *GT*: glycosyltransferase, *OMT*: O-methyltransferase, *MAT*: malonyltransferase. White (W), blue **(B)**, and purple (P) qingke grains at the grain developmental stage (I), grain color changing stage (II), and grain maturation stage (III), respectively. **(B)** LC−MS/MS analyses the activity of six candidate genes. The number 1-5 represent five anthocyanin standards: 1: Cya-3,5-*O*-diglu; 2: Cya 3-*O*-rut; 3: Cya 3-*O*-glu; 4: Cya 3-*O*-malglu; 5: Cya. **(C)** The MS/MS spectra (main fragments) for predicted product 6 (Peo-O-malglu). **(D)** Schematic summary of GT, MT, and OMT activities in the anthocyanin biosynthetic pathway. Cya, cyanidin; Cya 3-*O*-rut, cyanidin 3-*O*-rutinoside; Cya 3-*O*-glu, cyanidin 3-*O*-glucoside; Peo-*O*-malglu, peonidin *O*-malonylglucoside.

Based on correlation analyses between anthocyanins and genes (*r* > 0.6), screening for candidate genes involved in the anthocyanin modification pathway was performed. The results identified six candidate modifier enzymes that exhibited high expression in the grain color-changing or maturation stages for P accession (**Figure 5A**). For four glycosyltransferases, phylogenetic analysis showed that *HvGT1* and *HvGT2* were clustered with the *P14726.1*, *P16165.1* and *Zm3GlcT* genes, which confer anthocyanin 3-*O*-glycosyltransferase function. Other glycosyltransferases *HvGT3* and *HvGT4* have closer protein similarities with *OsUGT707*, which function as a flavonoid 3-*O*-glycosyltransferase (**Supplementary Figure 6**). Furthermore, *in vitro* enzymatic characterization of four genes demonstrated the involvement of *HvGT1* and *HvGT2* in catalyzing the formation of cyanidin 3-*O*-glucoside from cyanidin, *HvGT3* in the formation of cyanidin 3,5-*O*-glucoside from cyanidin 3-*O*-glucoside and *HvGT4* in the formation of cyanidin 3-*O*-rutinoside from cyanidin 3-*O*-glucoside. Another candidate, the malonyltransferase gene (*HvMaT*), shared 26.2% amino acid sequence similarity with *Dv3MaT* (GenBank accession no. AF489108), which is involved in anthocyanin malonylation. The encoding product of *HvMaT* displayed malonyl transfer activity with cyanidin 3-*O*-glucoside (**Figure 5B**). In addition, sequence alignment showed one candidate methyltransferase sharing 47.6% of its amino acid sequence with *ZRP4* (GenBank accession no. L14063.1), which was previously reported as *O*-methylation of suberin, phenylpropanoid precursors. The enzymatic function of this candidate confirmed the methyl-transferase activity of *HvOMT* for the conversion of cyanidin *O*-malonylglucoside to peonidin *O*-malonylglucoside (**Figures 5B, C**). An updated anthocyanin modification pathway based on the confirmation of six genes’ enzymatic activities confirmed the crucial role of modifier enzymes in the accumulation of anthocyanin derivations during qingke grain developmental stages (**Figure 5D**).

### A novel transcription factor regulating the anthocyanin biosynthesis pathway

**Figure 6:**
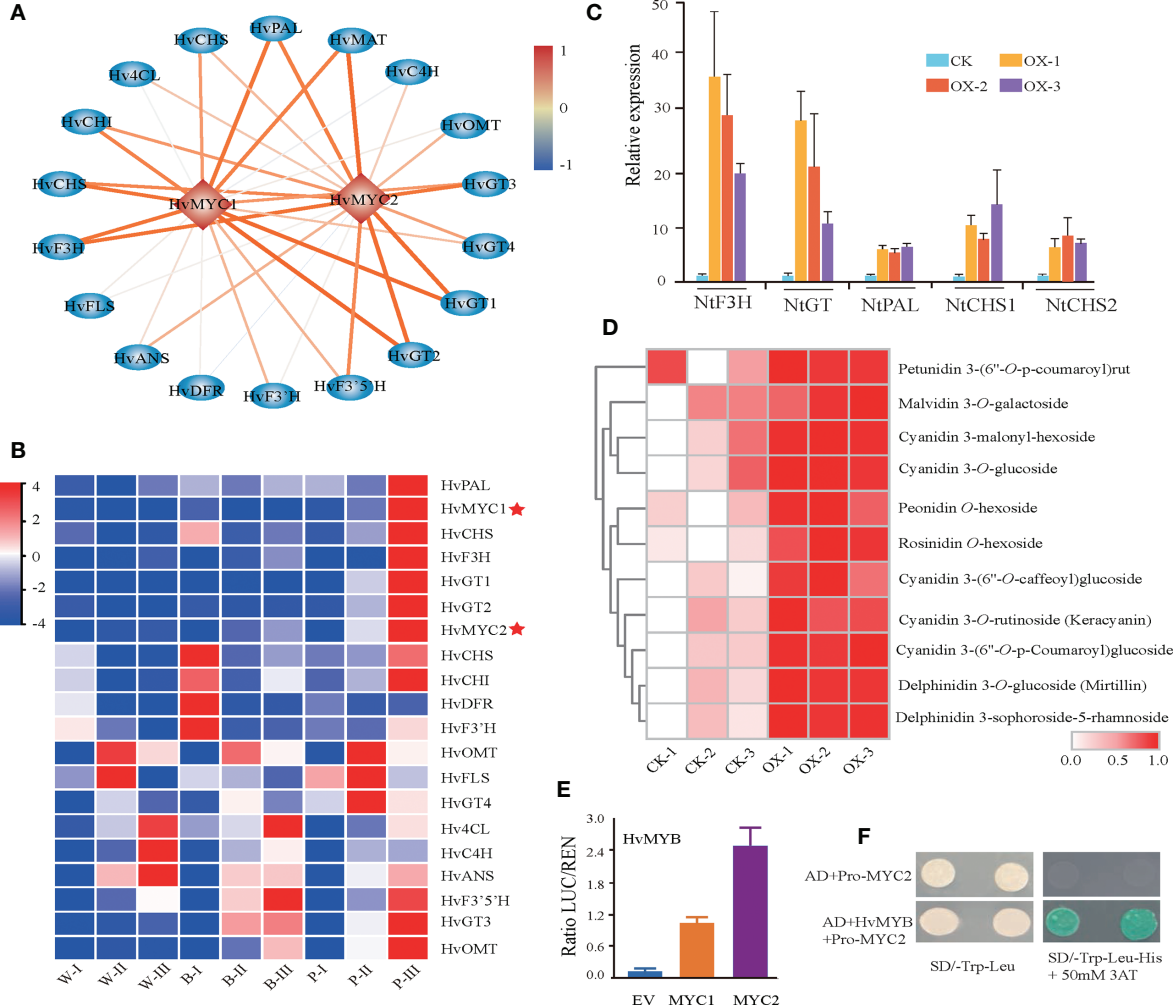
Identification of *HvMYC2* for the regulation of anthocyanin biosynthesis. **(A)** Transcriptional regulatory network constructed based on the correlation analysis of structural genes and TFs. The color scale from blue to red represents correlation values from -1 to 1. **(B)** Expression patterns of anthocyanin biosynthetic genes and TFs during the grain developmental stage. White (W), blue **(B)**, and purple (P) qingke grains at the grain developmental stage (I), grain color changing stage (II), and grain maturation stage (III), respectively. **(C, D)** Gene expression of anthocyanin-related genes **(C)** and anthocyanin **(D)** accumulation in tobacco leaves transiently overexpressing *HvMYC2*. CK-1, CK-2, and CK-3 represent the control (wild type). OX-1, OX-2, and OX-3 represent three different overexpression lines. **(E)** Two combinations of dual-luciferase reporters indicated that *HvMYB* has high binding to the promoter of *HvMYC2*. Data represent the means ± SDs. * represents a statistically significant difference (*t* test), *p* < 0.05 (one-way ANOVA, Tukey’s *post hoc* test). **(F)** *HvMYB* activates the *HvMYC2* promoter from qingke, as shown by the yeast one-hybrid assay.

Our coregulation network, in addition to the previously reported *HvMYC1*, also revealed a novel transcription factor, *HvMYC2*, which was strongly coexpressed with anthocyanin biosynthesis structural genes (**Figures 6A, B**). The Pearson correlation coefficient (PCC) (> 0.9) showed a high association of *HvMYC2* with structural genes, such as *HvF3H*, *HvGT1*, *HvGT2*, and *HvMAT* (**Supplementary Table 11**). To investigate the role of this *MYC* transcription factor, *HvMYC2* was transiently overexpressed in tobacco leaves and metabolite profiling analysis was conducted. Overexpression of *HvMYC2* in tobacco leaves lead to a clear overaccumulation of anthocyanins in the overexpression lines. In addition, we observed several anthocyanin biosynthesis-related genes in tobacco, for example, *NtF3H*, *NtPAL*, *NtCHS1* and *NtCHS2*, showed significant increased expression in the transient overexpression lines compared with control plants (**Figures 6C, D**). Further investigation revealed that *HvMYC2* could interact with the promoter of *HvF3H* to induce higher activity than that induced by *HvMYC1* based on dual-luciferase reporter assay (**Figure 6E**). Moreover, the yeast one-hybrid (Y1H) assay showed that *HvMYC2* directly binds to the *HvF3H* promoters (**Figure 6F**). In summary, our results that *HvMYC2* positively regulates the anthocyanin biosynthesis pathway in qingke.

To investigate whether *HvMYC2* is involved in the regulation and accumulation of anthocyanins in response to UV and cold stress, we selected purple (P1) and white (W1) qingke accessions to be treated with UV-B radiation and exposed to 4°C for 0, 1 and 24 h, respectively. qRT−PCR analysis confirmed that the expression levels of *HvMYC2* and the anthocyanin-related genes *HvPAL*, *HvF3H*, and *HvGT1* in leaves were significantly higher in the P1 accession than in the W1 accession under the two stress conditions. Furthermore, the metabolic analysis revealed that the accumulation of anthocyanins was markedly higher in P1 than in W1 under UV-B and cold stress. These results suggested the involvement of *HvMYC2* against UV and cold stress in colored qingke (**Supplementary Figure 7**).

Using the GMRN dataset, we constructed the global map of flavonoid metabolic changes during grain development process. In addition, our study not only confirmed the previously known metabolic regulatory networks but also identified the novel regulatory and structural genes that regulate or participate in the biosynthesis of anthocyanin in qingke (**Supplementary Figure 8**).

## Discussion

The primary goal of this study was to elucidate the mechanisms responsible for anthocyanin biosynthesis in qingke with different grain colors. For this purpose, metabolomic and transcriptomic analyses were performed on grains of three colored qingke varieties at different developmental stages.

Previous research has paid more attention to comparing grain tissues from various developmental phases using a single omics technique to provide a one-dimensional data analysis of transcriptional or biochemical changes (Peukert et al., 2014; Mahalingam, 2017; Bian et al., 2019). However, using a single omics technique is not recommended to accurately generate a comprehensive analysis of the metabolic regulatory network of the grain developmental process. In the present work, combining flavonoid metabolite and transcriptome analyses revealed a reprogramming of the flavonoid pathway with its flux diverted from isoflavone and flavonol branches toward the biosynthesis of downstream flavonoids, such as flavonoid C/O-glucoside. This result differs from previous studies where significant increases or decreases in flavonoid contents in Tartary buckwheat grains (Song et al., 2016; Li et al., 2019). A probable reason for this difference is the division of the entire flavonoid pathway into multiple branches, including flavonol, isoflavone, flavonoid and anthocyanin. For the flavonoid pathway, gene flux changes during grain development in rice, tartary buckwheat and sesame have already been studied (Huang et al., 2017; Wang et al., 2020). In these reports, *F3H*, *F3’H* and *UGT* from the downstream branch of the flavonoid pathway have shown upregulation for grain development stages. Our results are consistent with previous studies. In addition, our study also identified that, the enhancement of flavonoid metabolic flux is the key component to grain color formation in qingke, which has previously been studied in maize and peanut with remarkable accumulation of anthocyanins. However, unlike the colored seeds of other crops, such as maize and peanut, which only accumulate anthocyanins, we found that the flavonoid pathway precursors, intermediates, and derivatives also accumulated in the colored qingke seeds (Hu et al., 2016; Wan et al., 2020).

In different plant species, anthocyanin modifications (e.g., glycosylation, acylation, and methylation) have positively impacted their light-absorbing properties, stabilization in vacuoles or solution, and their bioavailability for health protection (Luo et al., 2007). The central biosynthetic pathway producing anthocyanin has been well studied previously (Koes et al., 2005). Previous studies have identified multiple anthocyanin biosynthesis genes (*PAL*, *C4H*, *4CL*, *CHS*, *CHI*, *F3H*, *F3’H*, *DFR*, *ANS*, *GT*, and *ACT*) showing the significantly higher expression in colored qingke, and their expression was strongly correlated with the content of anthocyanins (Yao et al., 2022). It is important to note that the initial steps to generate anthocyanin have been well-studied. However, the specific role of genes and their encoded enzymes responsible for anthocyanin modifications remain unclear. In the present study, we confirmed the functions of six key modifier enzymes that catalyze the glycosylation, malonylation, and methylation of anthocyanins. These three different types of enzymes can also be used in future as biocatalytic tools to synthesize bioactive anthocyanin modifications.

Previous studies using metabolite-based genome-wide association studies (mGWAS) have identified that, *HvMYC1* participates in anthocyanin biosynthesis and regulation in qingke grains (Zeng et al., 2020). In our study, *HvMYC1* was highly associated with flavonoid-metabolizing genes. Significantly higher expression correlations between *HvMYC2* and anthocyanin biosynthesis key genes, including *HvF3H*, *HvGT1*, *HvGT2* and *HvMAT*, helps us to speculate that *HvMYC2* may play a key role in the modulation of anthocyanin accumulation. Previous reports have shown that, the accumulation of anthocyanins plays an important role in increasing the plant tolerance against abiotic stress (Naing et al., 2017; Li et al., 2019a). In our study, UV-B and low temperature can significantly activated the expression of *HvMYC2* in white and purple qingke varieties. However, *HvMYC2* expression was significantly higher in purple qingke than in white qingke. Based on our results, we hypothesized that *HvMYC2* is responsible for the accumulation of anthocyanins and plays an important physiological role in protecting qingke against UV and cold stresses. Furthermore, our findings might make it possible to use *HvMYC2* to breed qingke varieties with high anthocyanin content and strong UV-B/low temperature adaptation.

## Conclusion

In this study, metabolomic and transcriptomic analyses have been carried out to investigate the grain coloration in qingke. The colored qingke grains had higher anthocyanin contents than the white qingke grains. We identified a core set of genes important for anthocyanin accumulation in qingke. Of these, *HvMYC2* was confirmed to be involved in anthocyanin biosynthesis. Based on correlation analyses between anthocyanins and genes, we further dissected the major anthocyanin modification pathway in colored qingke. These results improve our understanding of the anthocyanin accumulation and the underlying mechanism of anthocyanin biosynthesis in qingke grain and provide serious target genes for grain coloration to guide the improvement in qingke quality.

## Data availability statement

The original contributions presented in the study are publicly available. This data can be found here: NCBI, PRJNA797690.

## Author contributions

CX, HMKA, CZ, and YH contributed equally to this work. JL and XZ designed and supervised the project. HZY, YW, HY, and YH participated in the material preparation, phenotypic identification, and DNA and RNA extraction. CX, HMKA, CZ, SH, and YH conducted metabolic profiling and carried out the data analyses. CX wrote the manuscript. HMKA, CZ, JL, and XZ revised the manuscript. All authors contributed to the article and approved the submitted version.

## Funding

This research was supported by the following funding sources: Project supported by the Joint Funds of the National Natural Science Foundation of China (No.U20A2026), the Natural Science Foundation of Hainan Province (322RC574), the Hainan Yazhou Bay Seed Lab Postdoc Fund (B21Y10901 to CX), National Science Fund for Distinguished Young Scholars (No.31625021) and the Hainan University Startup Fund KYQD (ZR) 1866 to JL.

## Conflict of interest

The authors declare that the research was conducted in the absence of any commercial or financial relationships that could be construed as a potential conflict of interest.

## Publisher’s note

All claims expressed in this article are solely those of the authors and do not necessarily represent those of their affiliated organizations, or those of the publisher, the editors and the reviewers. Any product that may be evaluated in this article, or claim that may be made by its manufacturer, is not guaranteed or endorsed by the publisher.

## Supplementary material

Supplementary Figure 1 Dynamics of metabolite and gene expression during the grain developmental stages. *K*-means clustering grouped the transcriptome (blue) and metabolome (red) matrix profiles into ten clusters. The x-axis shows nine samples from three key grain developmental stages, and the y-axis represents the *Z* score per gene or metabolite. The numbers are shown in each subclass (for example, 531 genes and 17 metabolites for Cluster I) cluster from all metabolites and genes across all nine samples. White (W), blue (B), and purple (P) qingke grains at the grain developmental stage (I), grain color-changing stage (II), and grain maturation stage (III), respectively.

Supplementary Figure 2 KEGG pathways of the differentially expressed genes (DEGs) among the three colored qingke varieties at the grain developmental stage (I) **(A)**, grain color-changing stage (II) **(B)**, and grain maturation stage (III) **(C)**.

Supplementary Figure 3 Overview of transcriptome data analysis during grain development stages. **(A)** PCA of transcriptomic data from three developmental and nine samples in qingke. **(B)** Transcriptomic data were divided into two clades during grain development stages. Genes in Clade I are highly expressed during the first stage of grain development. Genes in Clade II are highly expressed during grain color-changing and mature stages. White (W), blue (B), and purple (P) qingke grains in the grain developmental stage (I), grain color-changing stage (II), and grain maturation stage (III), respectively.

Supplementary Figure 4 Expression levels of four genes related to flavonoid biosynthesis in RNA-seq (green) and validation by RT−qPCR (yellow). Error bars represent the standard deviation of three independent experiments. White (W), blue (B), and purple (P) qingke grains in the grain developmental stage (I), grain color-changing stage (II), and grain maturation stage (III), respectively.

Supplementary Figure 5 Correlation analysis of anthocyanin and gene expression during grain development as identified by WGCNA. **(A)** Dendrogram showing 13 coexpression modules constructed by hierarchical clusters. **(B)** Analysis of module-anthocyanin associations based on Pearson correlations. The red box indicates that the salmon module is strongly associated with the contents of four anthocyanins (*r* > 0.6, *p* < 0.01).

Supplementary Figure 6 Phylogenetic analysis of glycosyltransferase genes using MEGA. Bootstrap values > 70% from 1000 replications are indicated. The genes mentioned in the main text are as follows: *P14726.1* and *P16165.1* (Hall et al., 2011), *Zm3GlcT* (Veljanovski and Constabel, 2013), and *OsUGT707* (Peng et al., 2017).

Supplementary Figure 7 Expression of anthocyanin-related genes and the accumulation of anthocyanins analyze in two qingke varieties, P1 (purple qingke) and W1 (white qingke), under UV **(A)** and cold treatments **(B)**.

Supplementary Figure 8 Metabolic dynamics and regulatory network for flavonoid metabolites analyze in qingke. A regulatory network was constructed through a combination of flavonoid and transcriptome analyses to identify key transcription factors and their enzymes involved in the modification pathway.
