# Supplementary figures and images for: Integrative metabolomic and transcriptomic analyses reveal the mechanisms of Tibetan hulless barley grain coloration
Source: Front Plant Sci. 2022 Oct 25;13:1038625. doi: 10.3389/fpls.2022.1038625 (PMC9641248; doi:10.3389/fpls.2022.1038625)

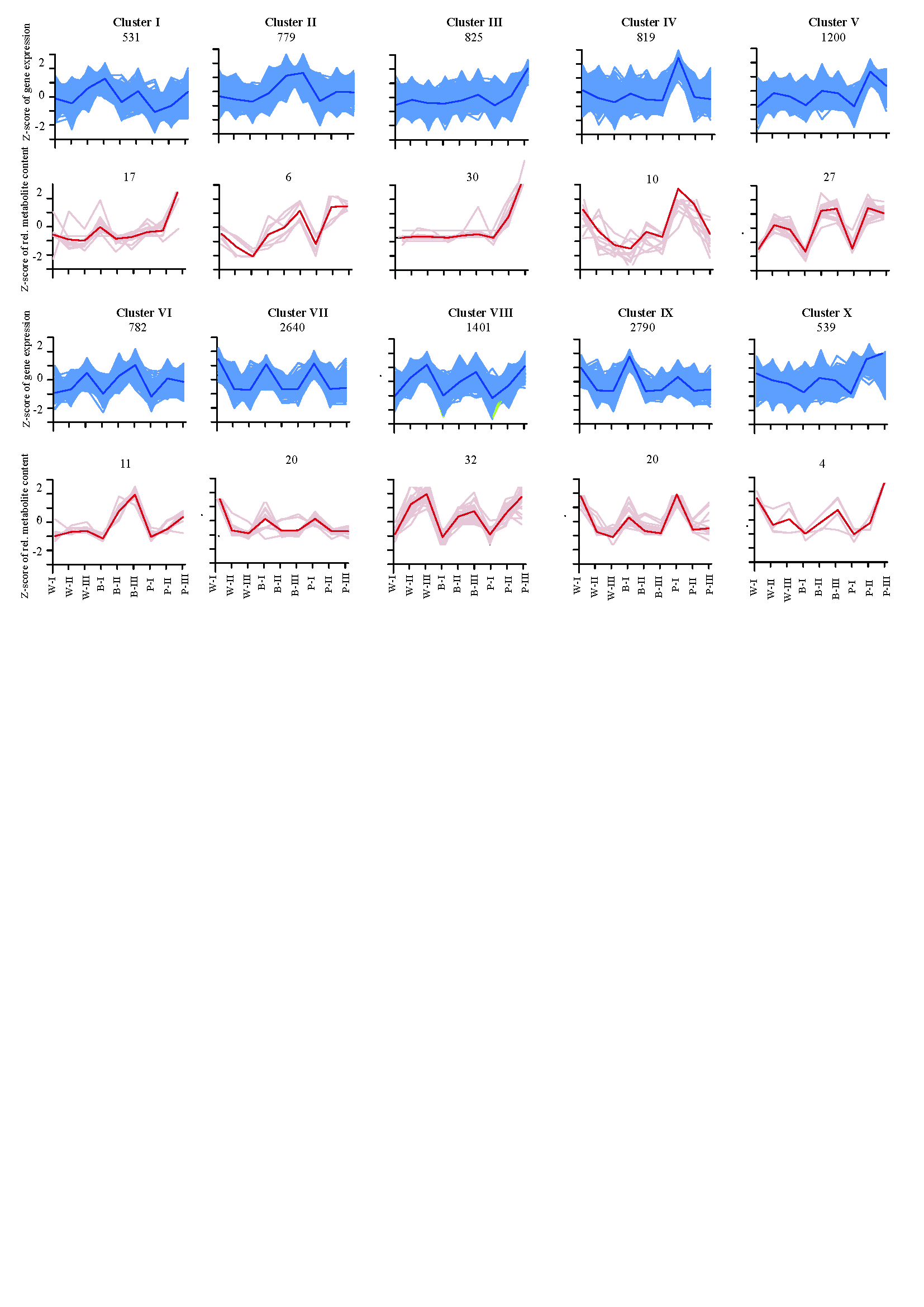

Supplement: Supplementary Figure 1 — Dynamics of metabolite and gene expression during the grain developmental stages. K-means clustering grouped the transcriptome (blue) and metabolome (red) matrix profiles into ten clusters. The x-axis shows nine samples from three key grain developmental stages, and the y-axis represents the Z score per gene or metabolite. The numbers are shown in each subclass (for example, 531 genes and 17 metabolites for Cluster I) cluster from all metabolites and genes across all nine samples. White (W), blue (B), and purple (P) qingke grains at the grain developmental stage (I), grain color-changing stage (II), and grain maturation stage (III), respectively. [file Image_1.tif]

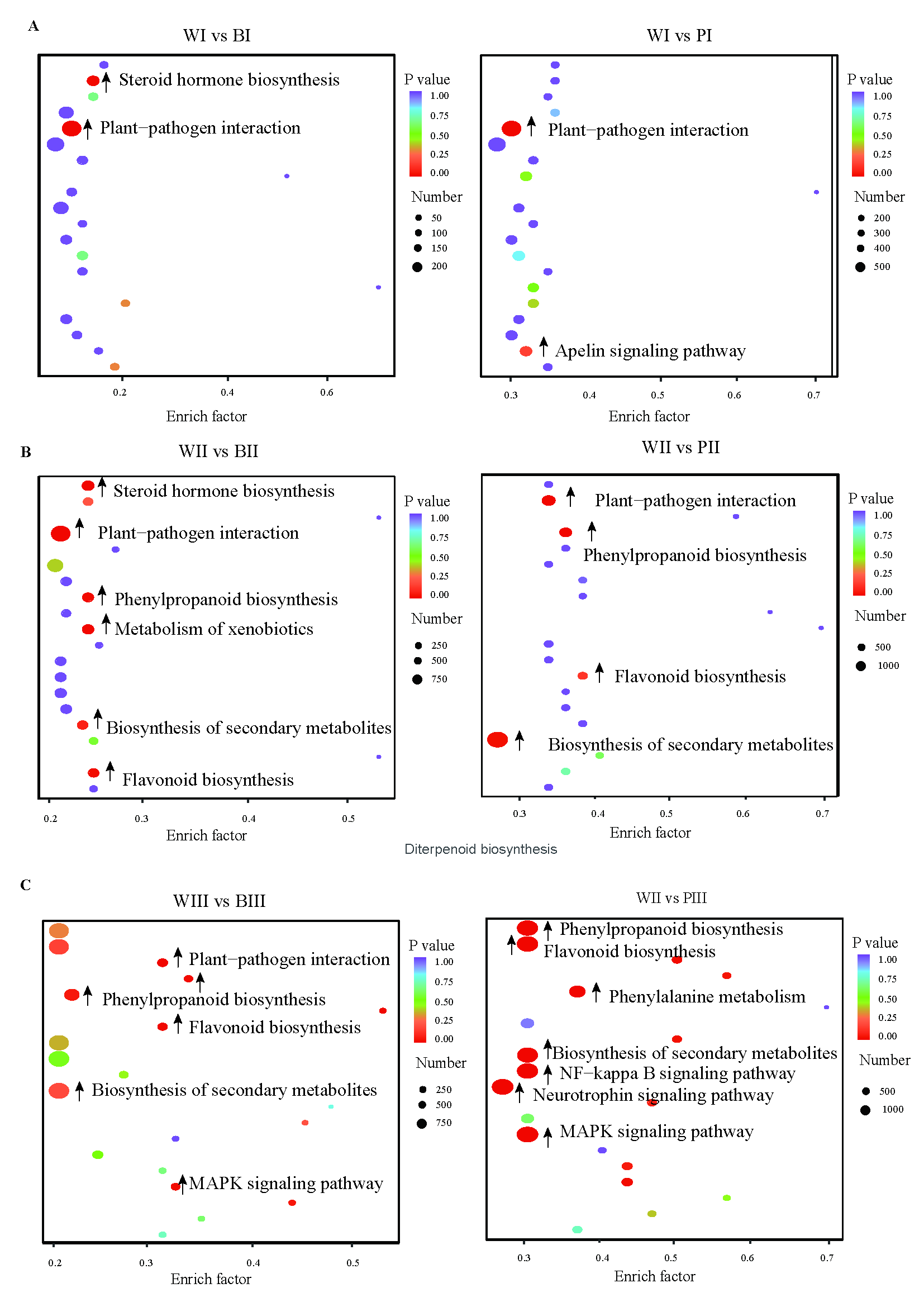

Supplement: Supplementary Figure 2 — KEGG pathways of the differentially expressed genes (DEGs) among the three colored qingke varieties at the grain developmental stage (I) (A), grain color-changing stage (II) (B), and grain maturation stage (III) (C). [file Image_2.tif]

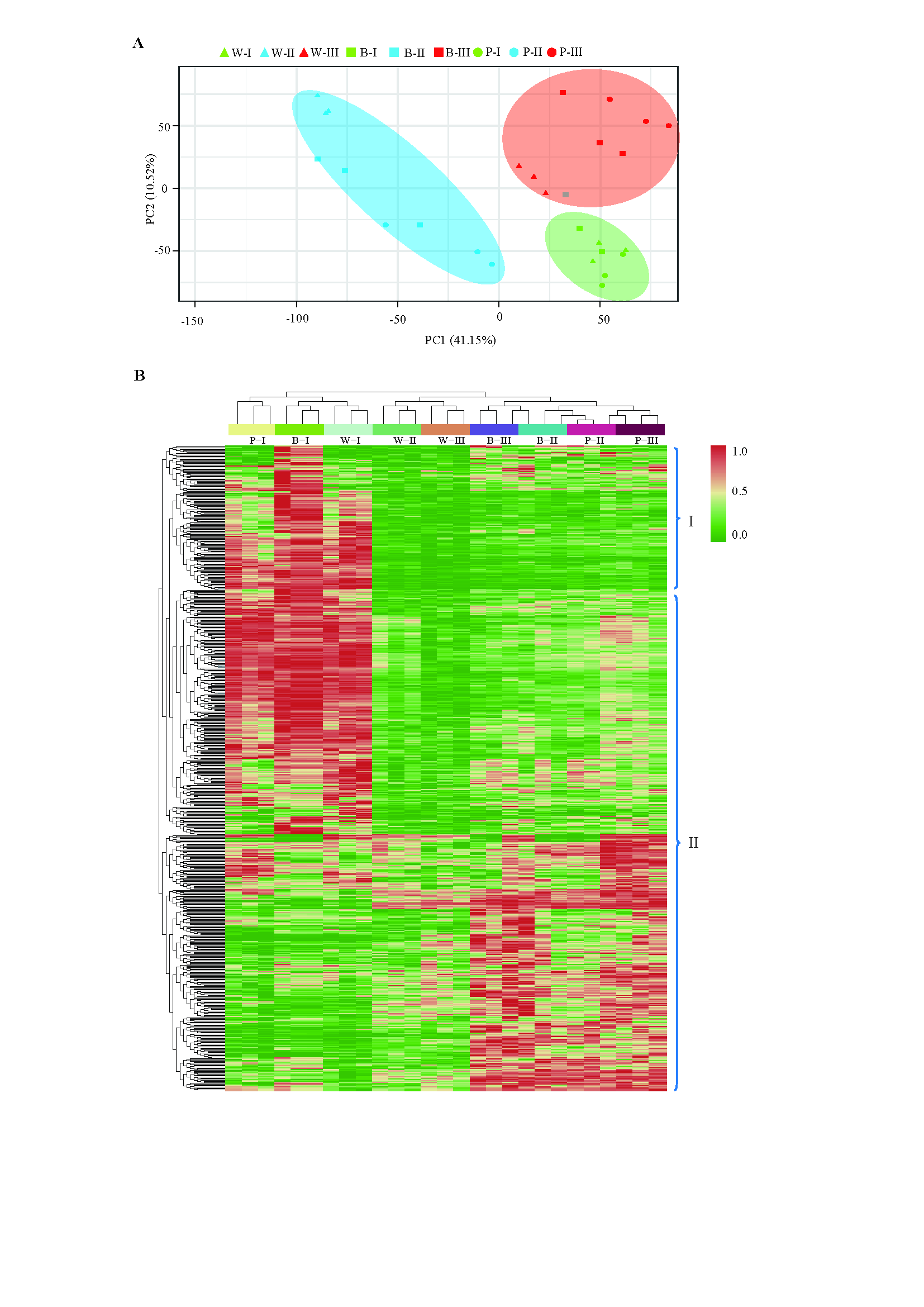

Supplement: Supplementary Figure 3 — Overview of transcriptome data analysis during grain development stages. (A) PCA of transcriptomic data from three developmental and nine samples in qingke. (B) Transcriptomic data were divided into two clades during grain development stages. Genes in Clade I are highly expressed during the first stage of grain development. Genes in Clade II are highly expressed during grain color-changing and mature stages. White (W), blue (B), and purple (P) qingke grains in the grain developmental stage (I), grain color-changing stage (II), and grain maturation stage (III), respectively. [file Image_3.tif]

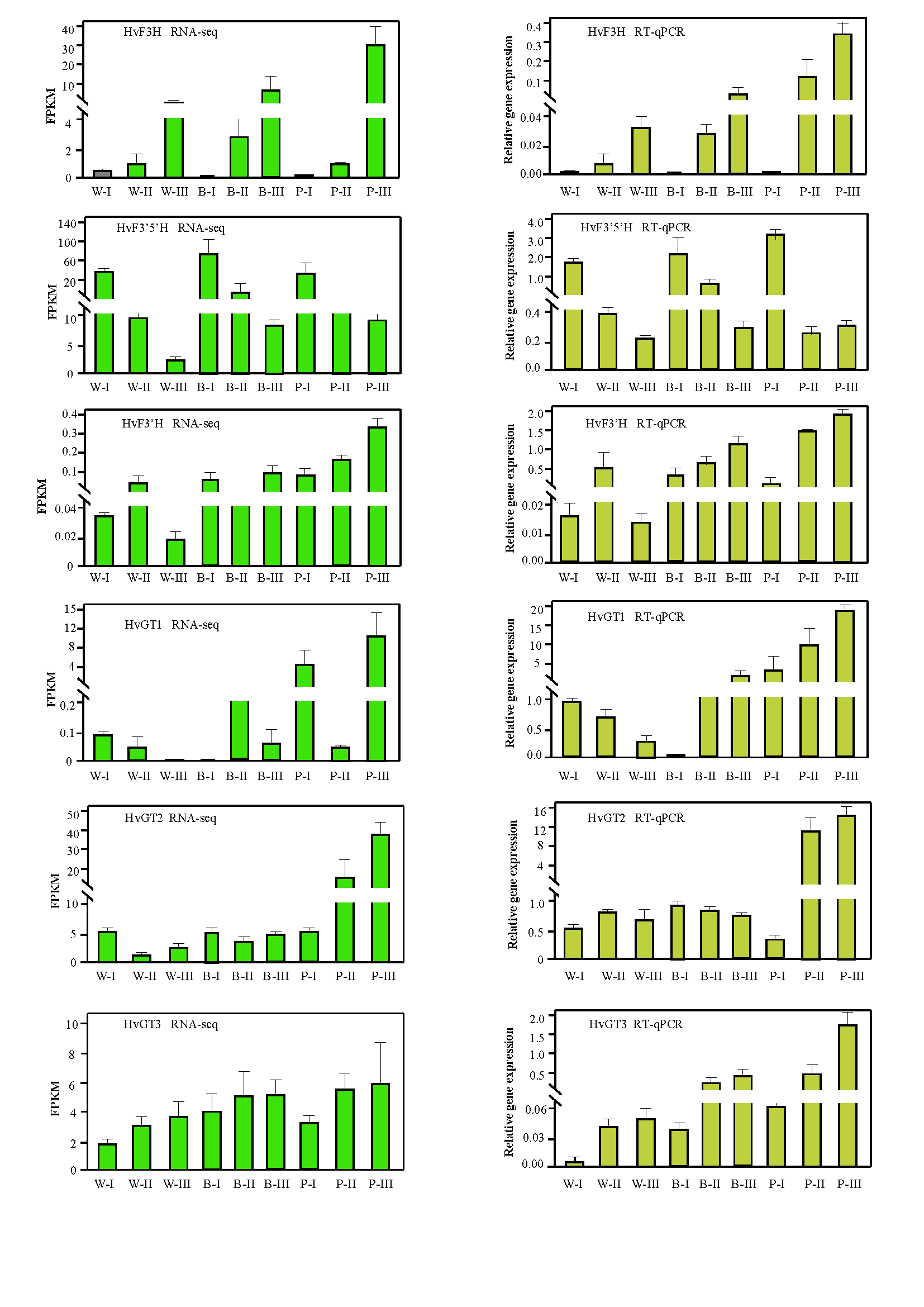

Supplement: Supplementary Figure 4 — Expression levels of four genes related to flavonoid biosynthesis in RNA-seq (green) and validation by RT−qPCR (yellow). Error bars represent the standard deviation of three independent experiments. White (W), blue (B), and purple (P) qingke grains in the grain developmental stage (I), grain color-changing stage (II), and grain maturation stage (III), respectively. [file Image_4.tif]

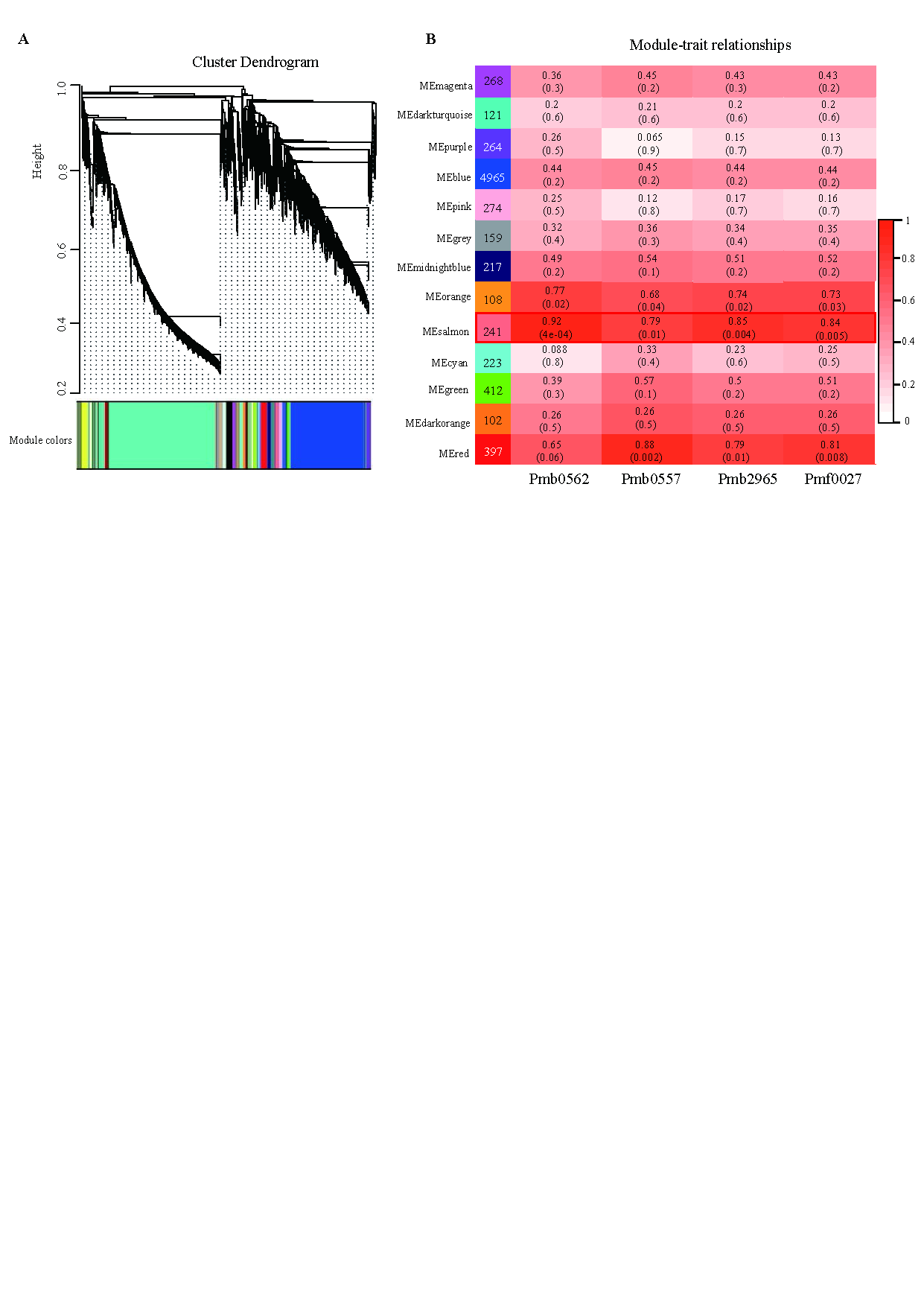

Supplement: Supplementary Figure 5 — Correlation analysis of anthocyanin and gene expression during grain development as identified by WGCNA. (A) Dendrogram showing 13 coexpression modules constructed by hierarchical clusters. (B) Analysis of module-anthocyanin associations based on Pearson correlations. The red box indicates that the salmon module is strongly associated with the contents of four anthocyanins (r > 0.6, p < 0.01). [file Image_5.tif]

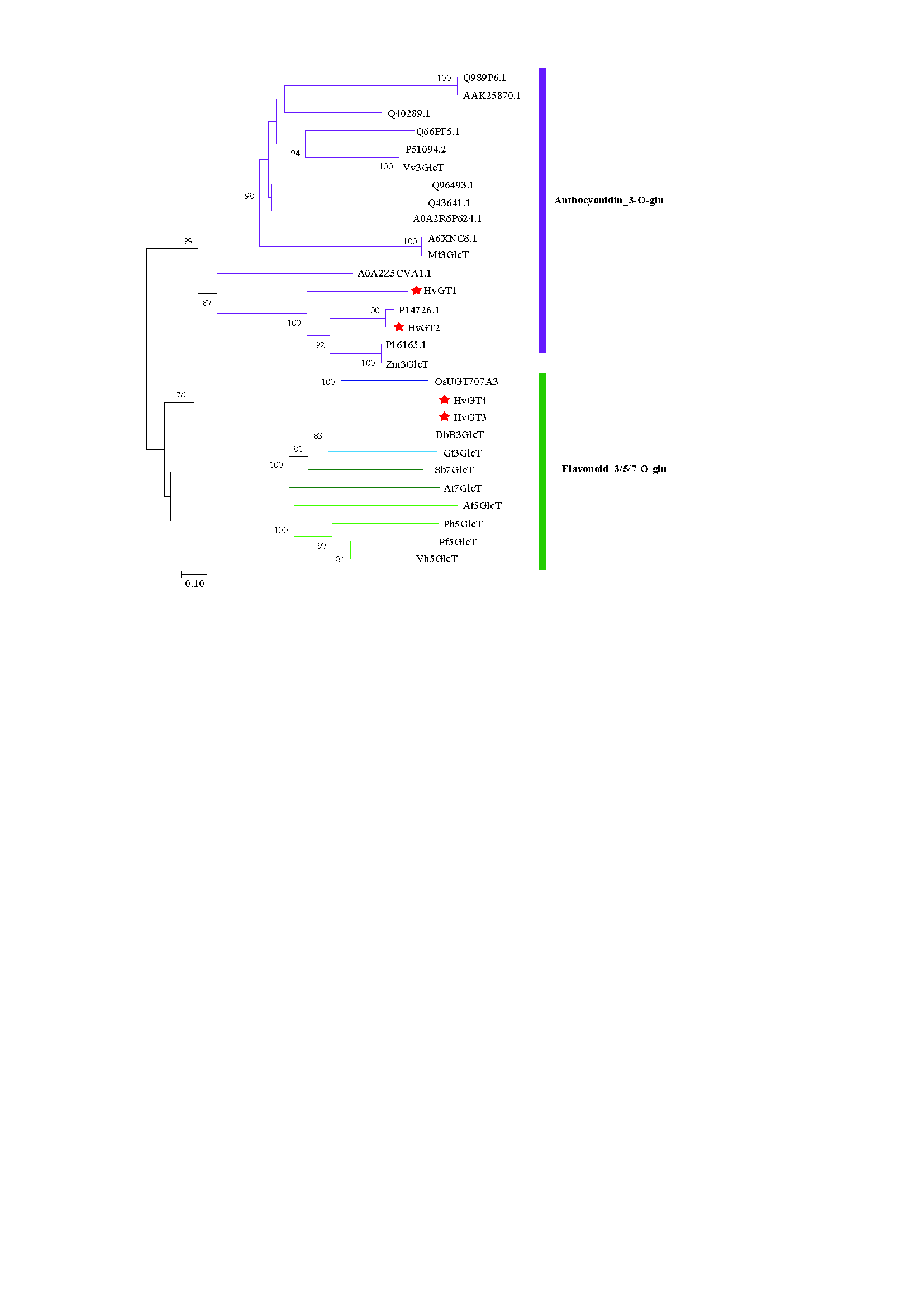

Supplement: Supplementary Figure 6 — Phylogenetic analysis of glycosyltransferase genes using MEGA. Bootstrap values > 70% from 1000 replications are indicated. The genes mentioned in the main text are as follows: P14726.1 and P16165.1 (Hall et al., 2011), Zm3GlcT (Veljanovski and Constabel, 2013), and OsUGT707 (Peng et al., 2017). [file Image_6.tif]

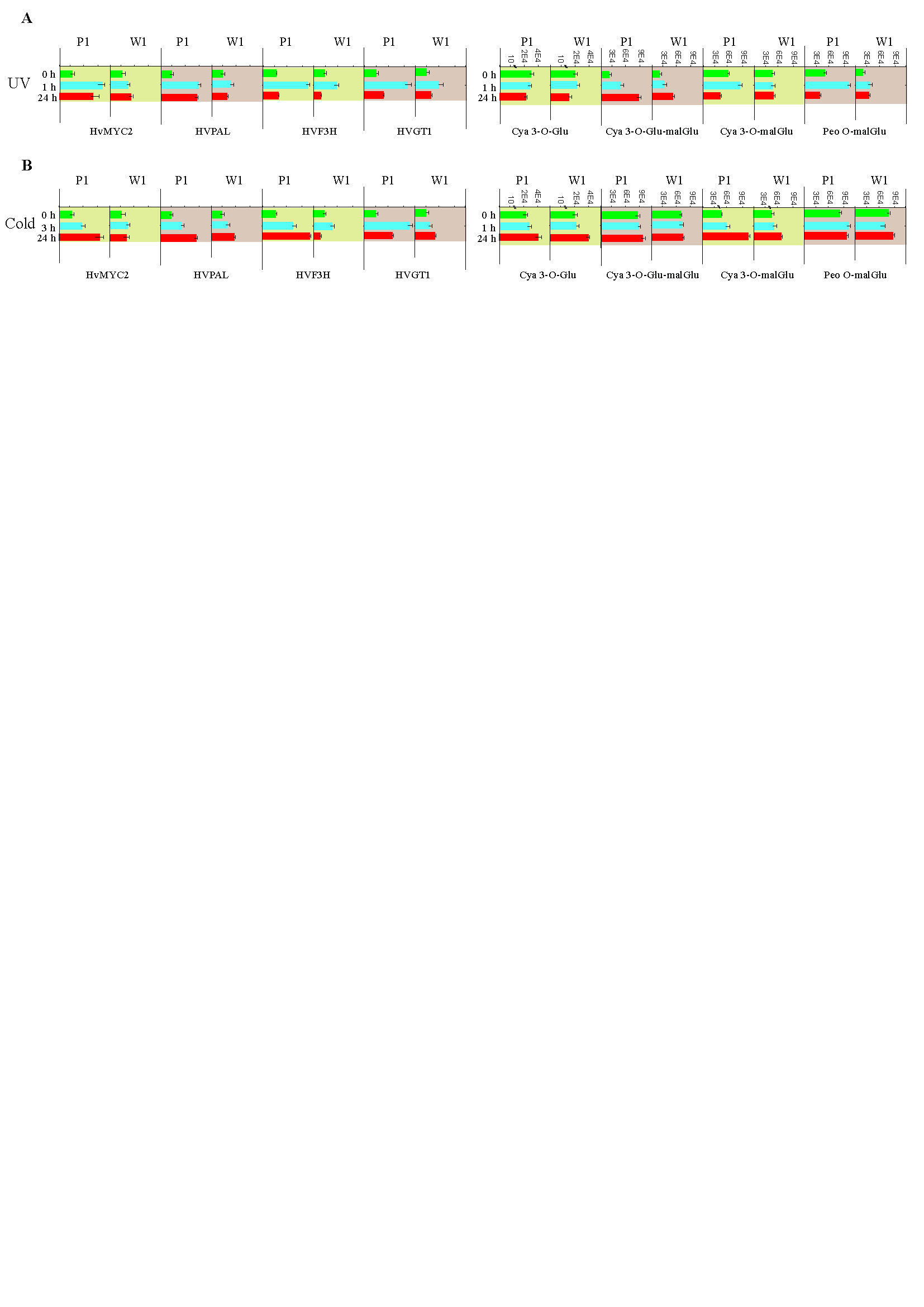

Supplement: Supplementary Figure 7 — Expression of anthocyanin-related genes and the accumulation of anthocyanins analyze in two qingke varieties, P1 (purple qingke) and W1 (white qingke), under UV (A) and cold treatments (B). [file Image_7.tif]

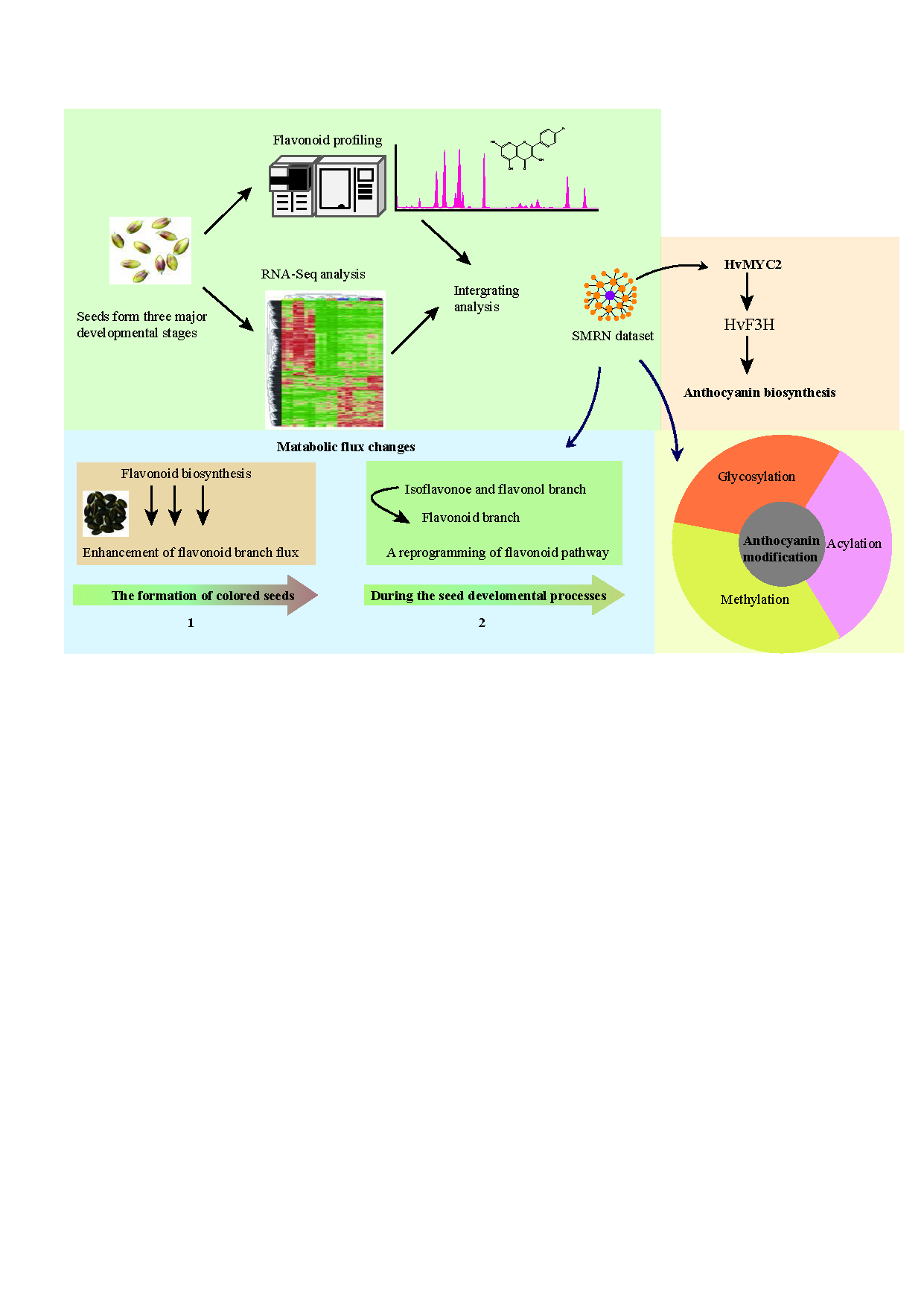

Supplement: Supplementary Figure 8 — Metabolic dynamics and regulatory network for flavonoid metabolites analyze in qingke. A regulatory network was constructed through a combination of flavonoid and transcriptome analyses to identify key transcription factors and their enzymes involved in the modification pathway. [file Image_8.tif]
